# Generalising better: Applying deep learning to integrate deleteriousness prediction scores for whole-exome SNV studies

**DOI:** 10.1371/journal.pone.0192829

**Published:** 2018-03-14

**Authors:** Ilia Korvigo, Andrey Afanasyev, Nikolay Romashchenko, Mikhail Skoblov

**Affiliations:** 1 Laboratory of Functional Analysis of the Genome, Moscow Institute of Physics and Technology, Moscow, Russia; 2 Laboratory of Microbiological Monitoring and Bioremediation of Soils, All-Russia Research Institute for Agricultural Microbiology, St. Petersburg, Russia; 3 iBinom Inc., Los Angeles, CA, United States of America; 4 Research Center for Medical Genetics, Moscow, Russia; 5 ITMO University, St. Petersburg, Russia; Cincinnati Children’s Hospital Medical Center, UNITED STATES

## Abstract

Many automatic classifiers were introduced to aid inference of phenotypical effects of uncategorised nsSNVs (nonsynonymous Single Nucleotide Variations) in theoretical and medical applications. Lately, several meta-estimators have been proposed that combine different predictors, such as PolyPhen and SIFT, to integrate more information in a single score. Although many advances have been made in feature design and machine learning algorithms used, the shortage of high-quality reference data along with the bias towards intensively studied *in vitro* models call for improved generalisation ability in order to further increase classification accuracy and handle records with insufficient data. Since a meta-estimator basically combines different scoring systems with highly complicated nonlinear relationships, we investigated how deep learning (supervised and unsupervised), which is particularly efficient at discovering hierarchies of features, can improve classification performance. While it is believed that one should only use deep learning for high-dimensional input spaces and other models (logistic regression, support vector machines, Bayesian classifiers, etc) for simpler inputs, we still believe that the ability of neural networks to discover intricate structure in highly heterogenous datasets can aid a meta-estimator. We compare the performance with various popular predictors, many of which are recommended by the American College of Medical Genetics and Genomics (ACMG), as well as available deep learning-based predictors. Thanks to hardware acceleration we were able to use a computationally expensive genetic algorithm to stochastically optimise hyper-parameters over many generations. Overfitting was hindered by noise injection and dropout, limiting coadaptation of hidden units. Although we stress that this work was not conceived as a tool comparison, but rather an exploration of the possibilities of deep learning application in ensemble scores, our results show that even relatively simple modern neural networks can significantly improve both prediction accuracy and coverage. We provide open-access to our finest model via the web-site: http://score.generesearch.ru/services/badmut/.

## Introduction

Single amino-acid variation (caused by nonsynonymous single nucleotide substitutions—nsSNVs) is a valuable source of information that can help us understand the fundamental features of protein evolution and function as well as uncover causative variants behind inherent health conditions and develop custom treatment strategies to maximise therapeutic efficiency. The dramatic increase in our capacity to cheaply sequence human exomes (the part of the genome comprised of exons) has brought enormous amounts of information on genetic variation in human populations, which clearly has great potential in both theoretical and medical applications. The later fuels research towards the integration of personal genetic data into medical practice. In fact, various companies are already pushing the technology into consumer market, though the means to simplify and streamline the downstream analyses are still in the infancy, and our ability to interpret variation in a phenotypically-sensible manner leaves a lot to be desired. Untangling the connections between variation and phenotypic traits remains one of the greatest challenges of functional genomics, because only a small fraction of possible variants have been thoroughly investigated and manually reviewed with respect to their fitness impact [1]. Thus, a lot of effort has been put into developing the means to infer possible damage of uncategorised nsSNVs by employing machine-learning. As a result, over the past decade many algorithms have been developed for predicting deleteriousness. In order to make predictions these tools encode variants using multiple quantitative and qualitative features, e.g. sequence homology [2], protein structure [3, 4] and evolutionary conservation [5, 6]. This diversity of scoring tools has led to the creation of dbNSFP [7–9], a regularly updated specialised database that accumulates predictions of various scores alongside genomic features for most of the possible variants in the human exome.

**Fig 1:**
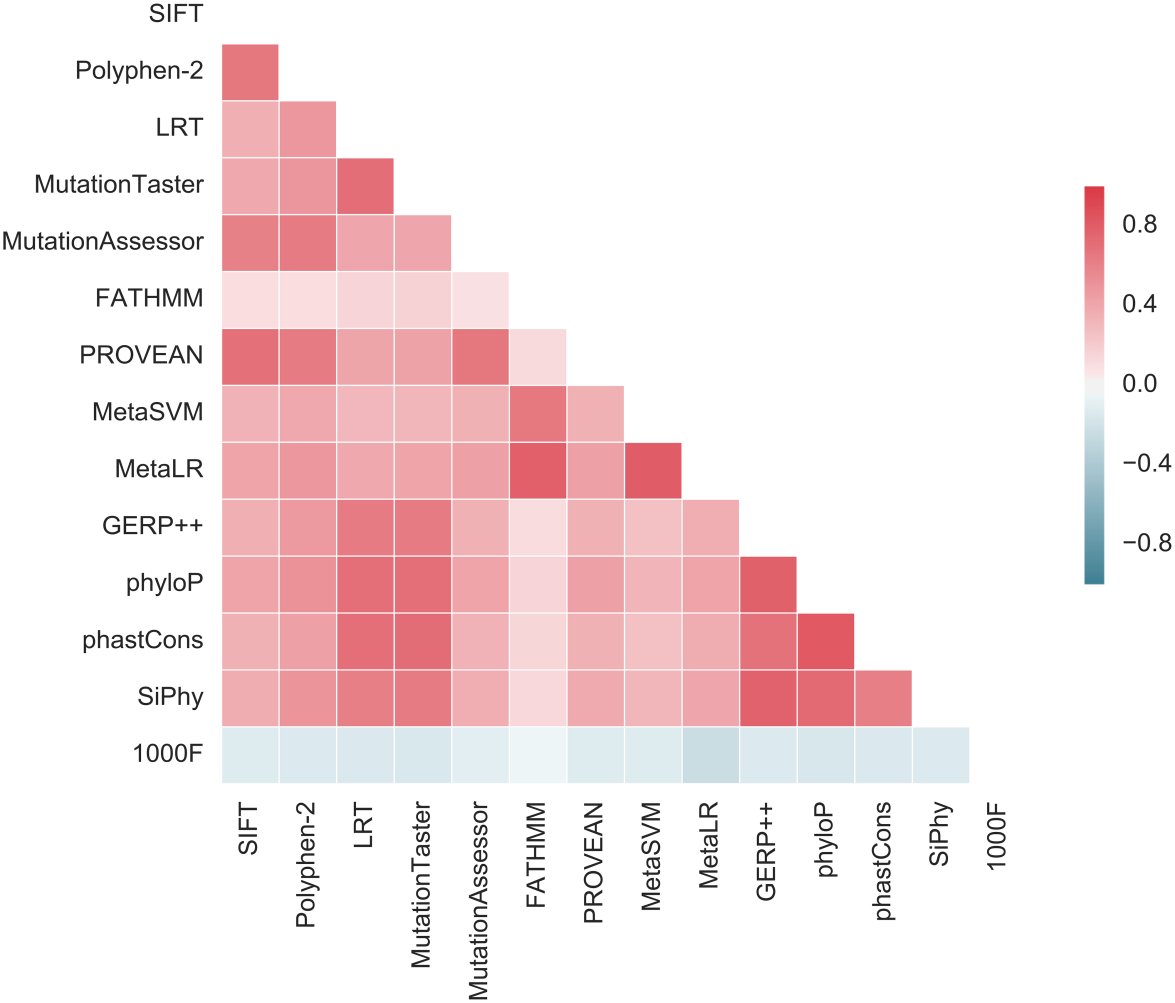
Prediction inconsistency. A heatmap of Spearman correlation between rank-transformed output values of different deleteriousness scoring systems. 1000F—allele frequency according to the 1000 Genomes project. Greater absolute correlation means greater consistency.

Meanwhile, the American College of Medical Genetics and Genomics (ACMG) published a guideline for reporting on clinical exomes [10], listing FATHMM [11], MutationAssessor [12], PANTHER [13], PhD-SNP [14], SIFT [15], SNPs&GO [16], MutationTaster [17], MutPred [18], PolyPhen-2 [19], PROVEAN [20], Condel [12], CADD [21], GERP [22], PhyloP [23] and several other scores as the most trustworthy. While the recommendations prove these scores useful, the guidelines describe them as merely accessory means of annotation, because differences in feature sets, training data and machine-learning algorithms used by the scores lead to inconsistent predictions (Fig 1), making the choice a matter of personal preference of each analyst [24].

While several extensive comparison studies have been carried out [24–26], the differences in benchmarking datasets, the number of tools and precision assessment methods further complicate the generalisability of their conclusions. Therefore, it is still unclear which tools to use for prioritising variants in exome-based studies of human diseases. To reduce bias, gather more available information and simplify tool selection several meta-estimators have been proposed, based on other scores, such as PolyPhen and Sift. It has been demonstrated that combining individual predictors in ensembles can be both effective and not. For example, KGGSeq (an ensemble of SIFT, PolyPhen-2, LRT, MutationTaster and PhyloP [27]), outperformed all scores it integrated in terms of ROC curve AUC (area under the curve), while CONDEL (another meta-estimator) failed to beat some its components [24]. Following the trend, the curators of dbNSFP have developed their own ensemble scores (MetaLR and MetaSVM), that outperform all widely used standalone scores and meta-scores [24]. Additionally, to overcome the shortage of reference data, crucial in purely supervised training, some authors have proposed unsupervised and semi-supervised learning strategies, with CADD being the most notable implementation of the idea, though it doesn’t peform well in benchmarks [24].

Missing predictions (missing feature values) pose another serious problem. When one or more of the tools used by a meta-score fails to process a substitution (e.g. due to lacking some information about it) the entry becomes incomplete (Table 1) and thus requires special handling. Some tools handle missing values like an intrinsic property of the data [21], some try to impute them (by basically adding another machine learning task) [24], others are restricted to complete entries.

**Table 1:** The fraction of nsSNVs with no predictions made by popular deleteriousness scores and the MetaLR meta-score.

Dataset	PolyPhen-2	SIFT	FATHMM	MutationTaster	MetaLR
Exome*	0.09	0.1	0.14	0.02	0.08
Test I**	0.02	0.03	0.06	0.01	0.004
Test II**	0.01	0.03	0.04	0.003	0.006

*The fractions are estimated by querying a random subset of 1 ⋅ 10^6^ SNVs from dbNSFP v3.2 [9].

**Our testing datasets I and II (described in the Materials and methods), comprising variations with experimental evidence of phenotype.

All these problems greatly emphasise the importance of better generalisation. Here we explore how deep learning can address the issues. Deep learning (DL) allows computational models learn hierarchies of representations with multiple levels of abstractions by combing several layers of nonlinear transformations. Techniques, such as noise injection and dropout, ultimately fight overfitting and allow adaptive regularisation [28]. Deep neural networks have already been used in DANN [29] and Eigen [30] to improve on the CADD’s original unsupervised approach, which incorporates hundreds of different features. While it is believed that one should only use DL for high-dimensional input spaces and other models (logistic regression, support vector machines, Bayesian classifiers, etc) for simpler inputs, we still believe that the ability of deep neural networks to discover intricate structure in highly heterogenous datasets can benefit a meta-estimator with relatively few input features, because connections and interaction between different scoring systems can be highly complicated and nonlinear [29]. We want to stress that this work was not conceived as a tool comparison, but rather an exploration of the possibilities of deep learning application in ensemble scores.

## Materials and methods

### Testing and training data

Our testing setup is based on the extensive comparative study performed by Dong et al. [24]. Since MetaLR and MetaSVM, introduced in the study, were shown to be state of the art in meta-estimators, it was natural to include them here for the sake of comparison along with other scores evaluated in that study. Thus we had to make sure that our training and testing data did not give our models an unfair advantage, hence we used the testing datasets provided by the authors. Briefly, the authors constructed their first testing dataset out of 120 deleterious mutations (causing 49 different diseases) recently reported in Nature Genetics, and 124 neutral mutations newly discovered from the CHARGE sequencing project [31]. To ensure the quality of the deleterious mutations, they only left variants reported to cause Mendelian diseases with experimental evidence. The quality of the neutral mutations was ensured by removing any record with minor allele frequency < 1% in 2 thousands exomes from the ARIC study via the CHARGE sequencing project [31]. Additionally the authors used a subset of VariBench protein tolerance dataset II [26]. VariBench, comprising high quality records with experimentally verified effects, has become a standard dataset for performance evaluation. The dataset itself contains 14611 positive and 19470 negative variants. The subset included 6279 deleterious curated variants and 13240 common neutral variants (minor allele frequency > 1%).

UniProtKB/Swiss-Prot was the main source of annotated nsSNVs for our training dataset. We downloaded all amino-acid natural variants (the HUMSAVAR archive from UniProt knowledge base release 03.2016) and mapped UniProt protein IDs to RefSeq nucleotide IDs. We then converted AA substitutions into nsSNVs. Initially there were 28 ⋅ 10^3^ causative and 39 ⋅ 10^3^ neutral AA polymorphisms. We then downloaded ClinVar variants mapped to loci referenced in OMIM [32]. Based on a dataset of 200 manually annotated records we trained a bag-of-words Naïve Bayesian classifier to automatically identify and remove SNVs associated with any type of cancer or having nothing but *in silico* and/or GWAS-based evidence of impact. This left us with around 120 ⋅ 10^3^ variants. We further filtered them to remove any possible splicing-altering substitutions using the annotations from SnpEff 4.1 [33]. Finally, we removed all SNVs yielding amino acid substitutions found in the testing datasets. After all these steps there were 96.5 ⋅ 10^3^ variants left: 64.5 ⋅ 10^3^ deleterious and 32 ⋅ 10^3^ neutral. This was our raw supervised training dataset. For our final supervised training collection we only left true positive records with experimental evidence. The dataset comprised around 19735 neutral and 14480 damaging nsSNVs S1 File. For our unsupervised training dataset we randomly sampled 10^6^ positions from the UCSC-annotated exonic regions. All data were collected for the hg19 genome assembly.

### Deep learning models

**Fig 2:**
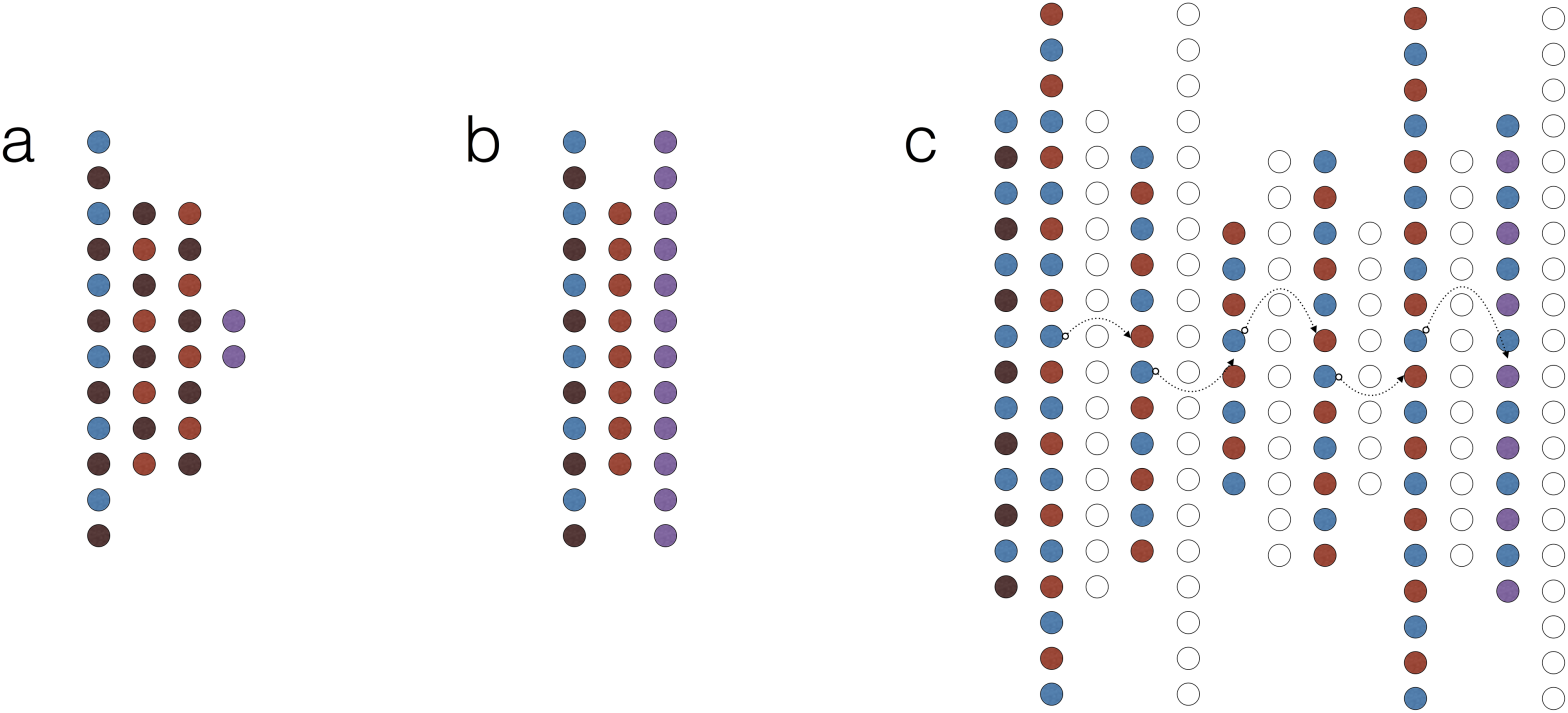
Network types. Schematic representation of basic deep learning models used in this study. (a) A multilayer perceptron (MLP). (b) A shallow denoising autoencoder (dAE). (c) Connecting dAEs into a stacked denoising autoencoder (sdAE); notice that each individual dAE learns to reconstruct the latent representation from the previous one (data stream is represented by arrows). Colours encode layer functions (combinations are possible): blue—input, light-red—latent, dark-red—dropout (noise), purple—output, hollow—discarded.

We constructed our classifiers using two basic architectures (Fig 2a): the deep multilayer perceptron (MLP) and the stacked denoising autoencoder (sdAE). MLPs are well known and widely used models. Although their basic architecture was introduced decades ago, their modern versions differ significantly in many implementation details. Stacked denoising autoencoders are relatively novel models used for unsupervised and semi-supervised learning and data compression. These networks are first trained as individual shallow denoising autoencoders (Fig 2b) by iteratively stacking one on top of another, which is followed by final training (Fig 2c). The term “denoising” stands for their ability to reconstruct lousy input records by generalising on training datasets. Stacking several autoencoders on top of each other and training each to reconstruct the output of the previous layer allows to learn a hierarchy of features in the input space in an unsupervised manner. When labeled reference data are scarce, one can combine unsupervised and supervised training to discover great generalisations from unlabelled data and perform fine-tuning using the few available labeled records.

### Implementation details

**Fig 3:**
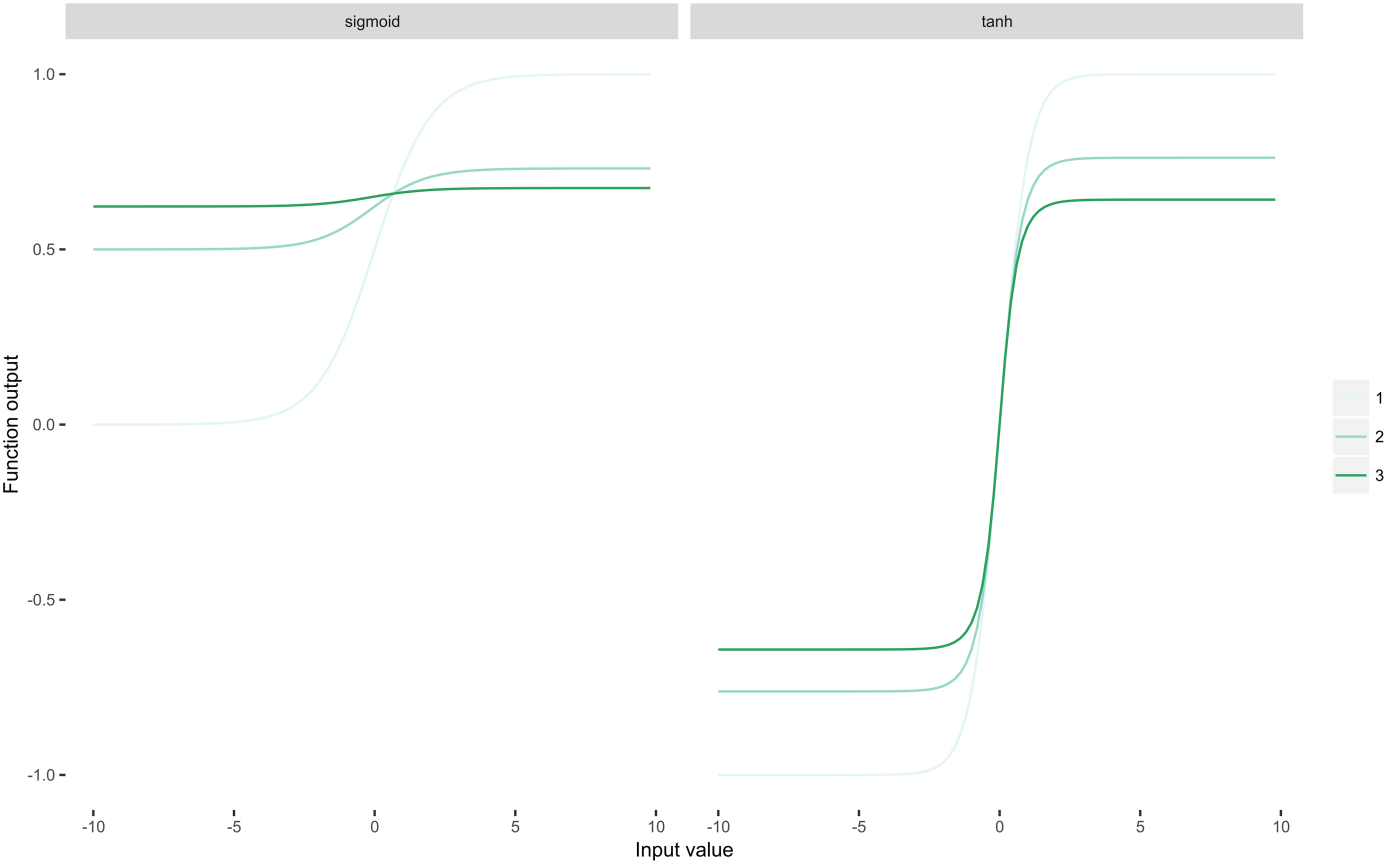
Nonlinearities. The sigmoid (a) and hyperbolic tangent (b) iteratively applied 3 times. Observe how repeated application of the sigmoid function quickly makes the gradient vanish completely.

Here we will briefly discuss several fundamental implementation details: update functions, regularisation, activation functions. Most neural networks are trained using various modifications of stochastic gradient descent (SGD). Here explored two SGD modifications: SGD with Nesterov momentum [34] and adagrad [35]. To prevent overfitting we used dropout as a simple and extremely effective regularisation tool [28, 36]. During training, dropout can be interpreted as sampling a part within the full network, and only updating the parameters of the subsampled units during back-propagation. In our MLPs we applied dropout to all layers, but the output; in sdAEs we only applied dropout to the input layer encouraging the networks to denoise the data. We used sigmoidal and ReLU (rectified linear unit) nonlinearities. Briefly, the standard sigmoid function is defined as $\sigma \left(x\right)=\frac{1}{1+\exp(-x)}$, hence it maps any real number into (0,1) and saturates at both ends, producing unfeasible gradients [37]. More importantly, repeated application of the sigmoid function (which basically happens in deep networks) leads to the vanishing gradient effect (Fig 3a) hindering convergence. We also used the hyperbolic tangent (tanh), which is considered a superior sigmoidal function, because it is zero-centered and less prone to the vanishing gradient effect (Fig 3b). The standard ReLU activation function is given by *ρ*(*x*) = *max*(0, *x*). It is idempotent (i.e. *ρ*°*ρ*°…°*ρ* = *ρ*) and scaling invariant (i.e. *ρ*(*αx*) = *αρ*(*x*)). These properties make it computationally cheap and immune to vanishing gradients [38]. At the same time, it has been shown tricky to use the function in autoencoders, due to knockout effect and overshooting [39], hence it is still more common to use sigmoidal activations in these models.

### Hyper-parameter optimisation and the training setup

So far we’ve mentioned various aspects of design and implementation, influencing performance in many different ways [37]. These settings are called hyper-parameters: the number of layers and units per each layer, the compression factor in encoders, learning rate, dropout and noise levels, mini-batch size, momentum applied, nonlinearities. To select these we used genetic algorithms (GA)—stochastic optimisation tools simulating natural selection over many generations of mutation, recombination and selective pressure [40]. This strategy has already been successfully applied to optimise hyper-parameters in other machine-learning models [41]. We performed two independent GA runs: one for the pure MLP model and one for the stacked denoising autoencoder. In both cases a population of 100 individuals had been evolving for 100 generations and each model could chose whether to use the 7-way or 100-way phyloP and phastCons conservation scores, the batch size (500-10000), adagrad or Nesterov momentum (0.00-1.0; step size 0.05) update functions and the learning rate (0.005-1.0).

During the MLP GA run the number of training epochs was fixed at 1000. All models used the hard ReLU activation function and Glorot uniform weight initialisation. Variable hyper-parameters:

- the number of hidden layers: 1-4
- the number of units per hidden layer: 10-30
- dropout probability: 0.00-0.5 (stepsize 0.05)

Each stacked denoising autoencoder trained in two steps: individual shallow autoencoders trained for 300 epochs prior stacking. Stacked autoencoders trained for additional 1000 epochs. We increased the number of training epochs due to the saturation and vanishing gradient problems inherent to sigmoidal nonlinearities. Hyper-parameter search space:

- first-layer expansion factor: 1.0-1.5 (stepsize 0.05); represents the relative increase in the number of units in the first hidden layer with respect to the input layer
- encoder compression level: 1.0-1.5 (stepsize 0.05)
- the number of hidden layers in the encoder (excluding the compressed latent layer): 1-3 (and the decoder by extension, due to symmetric design).
- activation function: sigmoid or hyperbolic tangent (in conjunction with appropriate weight initialisation functions).

We carried out the process on a machine with 8 Nvidia Titan X (Maxwell) GPUs (Graphics Processing Units) using model-based parallelism [42], i.e. each model trained on a separate GPU with its own copy of the data, hence we could train up to 8 models simultaneously. To estimate fitness we used 3-fold cross-validation scores (categorical crossentropy for MLPs, and squared-root reconstruction error for sdAEs). Neural networks were implemented using Theano and lasagne in Python 3.5. We used the genetic algorithm implementation provided by package *genetic*, openly available in PyPI (the Python Package Index).

### Data extraction and missing annotations

We selected the following scores and genomic features as input units: FATHMM, GERP++, LRT, LRT Omega, MetaLR, MetaSVM, MutationAssessor, MutationTaster, PROVEAN, Polyphen2 (both HDIV and HVAR), SIFT, SiPhy log Odds, phastCons vertebrate (both 7-way and 100-way), phyloP vertebrate (both 7-way and 100-way) and the allele frequency (AF) in the 1000 Genomes Project dataset. Since all these scores had different output scales and thus couldn’t be directly compared, we used the rank-transformed values, provided by dbNSFP [7], for both training and comparison like demonstrated by Dong et al. [24]. Although this step was only mandatory to train our autoencoders, other networks should have benefitted from the normalised data as well. For each position in the training and testing datasets we extracted these features from dbNSFP 3.1 and 3.2 [9] (we used the former to obtain values for the 7-way phyloP and phastCons, replaced by updated 100-way scores in the recent versions of dbNSFP). We had no special way of handling missing data: missing values in the training and testing datasets were simply replaced by zeros.

### Performance estimations

To make comparisons between different scores possible we used rank-transformed [7] outputs of CADD, DANN, Eigen, FATHMM, GERP++, LRT, MutationAssessor, MutationTaster, PROVEAN, Polyphen-2 (both HDIV and HVAR), SIFT, SiPhy (29-way), phastCons (100-way), phyloP (100-way). We interpreted these values as positive-class (causative) probabilities and carried out two series of benchmarks. The first one comprised threshold-invariant performance indicators: (1) the area under the receiver operating characteristic curve (ROC-curve AUC) and (2) the area under the precision-recall curve (average precision). The second one comprised cutoff-sensitive statistics: (1) the F1 score, (2) the Matthews correlation coefficient (MCC) and (3) the accuracy score. We optimised the cutoff value for each score individually to find the highest possible performance using empirical bootstrapping (1000 replicates) to approximate the distributions of these statistics and estimate their 95% confidence intervals (i.e. the 2.5 and 97.5 percentiles of the distributions). We benchmarked our classifiers (MLP and sdAE) without removing variations with missing annotations (i.e. incomplete data) from the dataset. In our interpretations we considered the second testing dataset more representative, because of its significantly greater size (∼100 times more records).

### Assessing generalisation

Since available testing data comprise a small subset of the exome, to extrapolate a classifier’s performance from these datasets to the entire exome, it is important to evaluate how representative the datasets are and to examine the classifier’s ability to generalise. We used Gene Ontology (GO) [43] terms to encode various protein properties and to analyse their distribution in the exome and the datasets. Since raw GO annotation was extremely sparse and deep, we mapped it onto the generic GO Slim annotation, reducing the GO term-space to ∼150 terms and making it shallow. This allowed us to include all term levels. We carried out binomial tests for each term to find the number of terms significantly enriched in either misclassified or correctly classified subsets of the datasets. Additionally, using the same procedure we tested term enrichment in the false-positive (FP) and false-negative (FN) subsets of the misclassified variations. We adjusted p-values using the Benjamini-Hochberg procedure, also known as the false discovery rate (FDR). For each predictor we used a probability cutoff-value maximising the F1 score. The FDR level was set to 5%.

## Results and discussion

### Training logs

We carried out two independent runs of the genetic algorithm to optimise the hyper-parameters in our deep learning models. The MLP run took 3 days of calculations. We selected five sets of parameters yielding the highest cross-validation scores and trained them for 50 ⋅ 10^3^ epochs. We then picked the network with the highest ROC-curve AUC and average precision (area under the precision-recall curve). The network had the following parameters:

- two latent layers: the first one had 13 hidden units, and the second one had 19
- dropout probability: 0.1
- batch size: 2000
- learning rate: 0.01
- update function: Nesterov momentum (0.7)
- phyloP and phastCons version: 7-way

The sdAE run took 54 days of calculations. As with the MLPs, we took 5 best-scoring models, though this time we trained each one for 100 ⋅ 10^3^ epochs. After benchmarking the models on one million random nsSNVs from the exome (non-overlapping with the training dataset), we picked one model with the lowest absolute-error of reconstruction. It had the following parameters:

- three latent layers in the encoder
- expansion factor: 1.25
- compression factor: 1.3
- input-layer dropout (noise) probability: 0.3
- batch size: 5000
- learning rate: 0.05
- update function: Nesterov momentum (0.5)
- nonlinearity: hyperbolic tangent
- phyloP and phastCons version: 100-way

This model achieved median absolute reconstruction error of 0.02. We then removed the decoding part of the model, added a softmax output layer with two units and trained the model for 10 ⋅ 10^3^ epochs to classify nsSNVs using the supervised training dataset. Training parameters were not altered, except for the batch size, which was reduced to 2000. Surprisingly, the resulting classifier performed poorly with average precision of 0.63 and ROC-curve AUC of 0.82, while having extremely low training errors, which led us to conclude that overfitting was the reason behind these results. To test this assumption, we tried to train the model again (staring with the same unmodified sdAE) while freezing the weights in the encoder and only updating the classifier’s softmax layer, which is basically similar to applying logistic regression on the compressed latent representation of the input space. This significantly increased both measures of performance. We used this final model in our benchmarks.

### Performance

**Fig 4:**
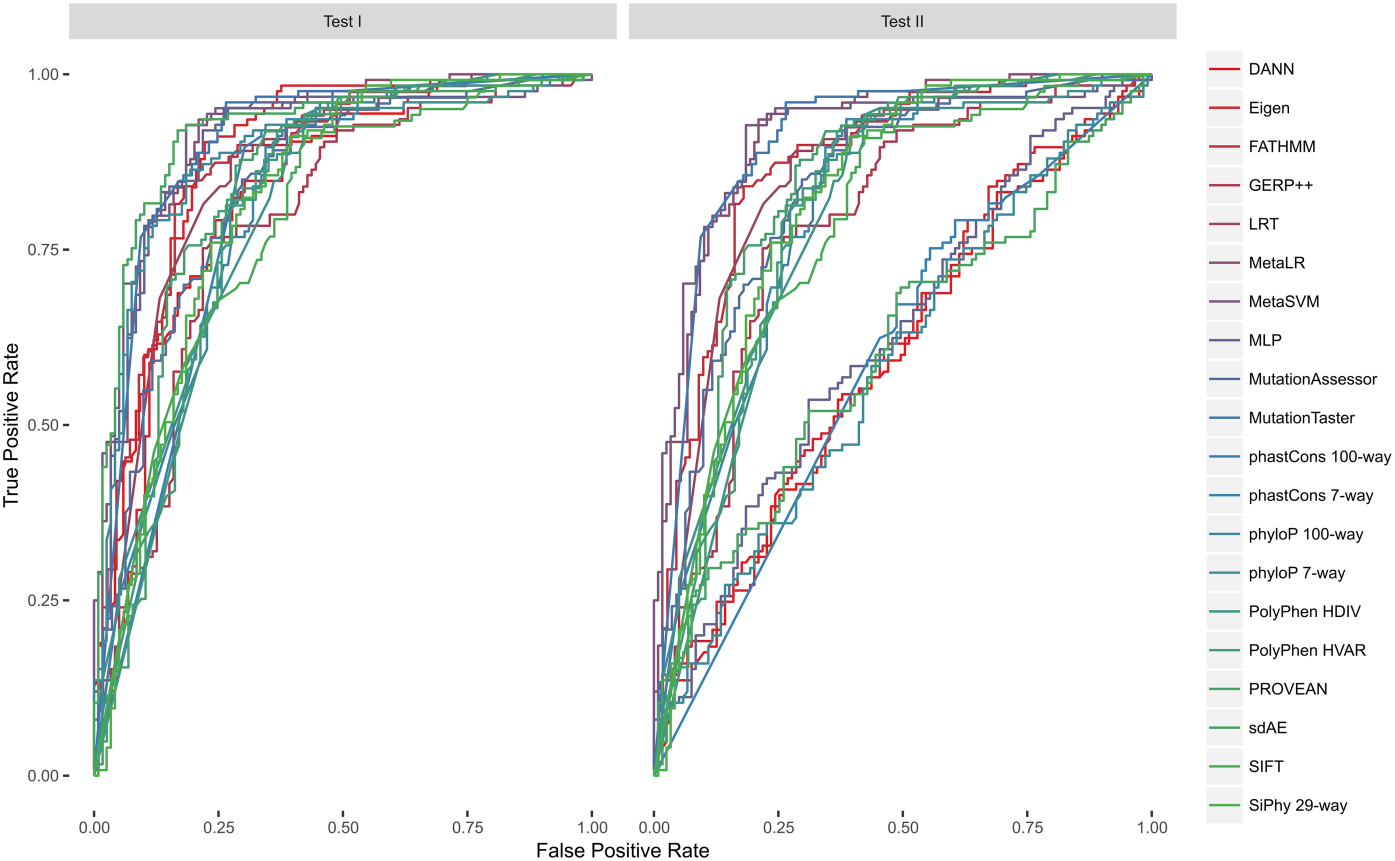
ROC-curves. MLP, MetaLR, MetaSVM, sDAE and MutationTaster produced the largest area under the curve.

The first round of benchmarks comprised cutoff-invariant performance measures: the ROC curve AUC and average accuracy score (the area under the precision-recall curve. The ROC curve AUC tests supported the results published by Dong et al. [24] in their comparative study (Table 2). The meta-estimators, introduced in that study (MetaLR and MetaSVM), outperformed most of the scores we used in the benchmark. Only our MLP classifier had a slight edge over both these scores in terms of the ROC curve AUC. Though, MLP and MetaLR showed identical performance on the test I, which was second only to MutationTater and sdAE, the MLP outperformed all the other scores on the test II. At the same time the stacked autoencoder outperformed all scores on the test I. Surprisingly enough, the deep learning models that were developed to improve on the CADD’s unsupervised approach (DANN and Eigen) performed worse than CADD itself. We also plotted the curves for better illustration (Fig 4).

**Table 2:** ROC curve AUC score with 95% confidence intervals.

Score	Test I	Test II
CADD	0.85 (0.79–0.90)	0.78 (0.78–0.79)
DANN	0.84 (0.79–0.89)	0.75 (0.74–0.75)
Eigen	0.86 (0.81–0.91)	0.67 (0.66–0.68)
FATHMM	0.84 (0.78–0.89)	0.91 (0.90–0.91)
GERP++	0.79 (0.73–0.84)	0.68 (0.67–0.68)
LRT	0.85 (0.80–0.90)	0.73 (0.72–0.74)
**MLP**	0.90 (0.86–0.94)	0.94 (0.94–0.95)
MetaLR	0.90 (0.86–0.94)	0.93 (0.93–0.94)
MetaSVM	0.90 (0.86–0.94)	0.92 (0.92–0.93)
MutationAssessor	0.78 (0.71–0.83)	0.77 (0.77–0.78)
MutationTaster	0.91 (0.87–0.94)	0.76 (0.75–0.77)
PROVEAN	0.82 (0.77–0.88)	0.77 (0.77–0.78)
PolyPhen HDIV	0.79 (0.73–0.85)	0.77 (0.76–0.77)
PolyPhen HVAR	0.80 (0.74–0.86)	0.79 (0.78–0.79)
SIFT	0.77 (0.71–0.82)	0.78 (0.77–0.78)
SiPhy 29-way	0.82 (0.76–0.87)	0.70 (0.70–0.71)
phastCons 100-way	0.81 (0.76–0.86)	0.69 (0.69–0.70)
phyloP 100-way	0.89 (0.85–0.93)	0.75 (0.74–0.76)
**sdAE**	0.92 (0.88–0.95)	0.92 (0.92–0.93)

While ROC curve AUC results gave our MLP a slight edge over the other scores on the test II, the average precision score deemed it even more superior (Table 3), though it was second to sdAE on the test I. In general, most scores fared better on the testing dataset I in case of both performance indicators.

**Table 3:** Average precision score with 95% confidence intervals.

Score	Test I	Test II
CADD	0.79 (0.70–0.87)	0.60 (0.58–0.61)
DANN	0.84 (0.77–0.90)	0.55 (0.53–0.56)
Eigen	0.81 (0.73–0.89)	0.57 (0.56–0.58)
FATHMM	0.83 (0.75–0.90)	0.83 (0.82–0.84)
GERP++	0.77 (0.69–0.85)	0.44 (0.43–0.45)
LRT	0.87 (0.82–0.92)	0.64 (0.63–0.65)
**MLP**	0.91 (0.82–0.94)	0.89 (0.88–0.91)
MetaLR	0.88 (0.81–0.94)	0.87 (0.87–0.89)
MetaSVM	0.91 (0.87–0.95)	0.87 (0.86–0.88)
MutationAssessor	0.78 (0.70–0.85)	0.67 (0.66–0.68)
MutationTaster	0.91 (0.87–0.95)	0.67 (0.67–0.68)
PROVEAN	0.76 (0.67–0.85)	0.61 (0.59–0.62)
PolyPhen HDIV	0.80 (0.73–0.86)	0.67 (0.66–0.68)
PolyPhen HVAR	0.77 (0.69–0.84)	0.66 (0.65–0.68)
SIFT	0.79 (0.72–0.85)	0.67 (0.66–0.68)
SiPhy 29-way	0.75 (0.65–0.84)	0.47 (0.45–0.48)
phastCons 100-way	0.86 (0.81–0.90)	0.66 (0.66–0.67)
phyloP 100-way	0.89 (0.83–0.94)	0.56 (0.54–0.57)
**sdAE**	0.92 (0.86–0.96)	0.87 (0.86–0.87)

The second round of benchmarks comprised three threshold-sensitive metrics: the F1-score, MCC and the weighted accuracy score (Table 4). Since we have optimised each score’s cutoff individually for each performance metric these results can be used a guideline for cutoff selection. For the most part all three metrics yield similar performance ranking. Once again, the MLP comes first with a slight edge over the MetaLR, MetaSVM and sdAE. The latter three show almost identical performance.

**Table 4:** Maximum average values of threshold-sensitive performance measures, evaluated for test II. Numbers in parentheses represent corresponding cutoffs.

Score	F1-score	MCC	Accuracy
CADD	0.64 (0.50)	0.43 (0.58)	0.74 (0.73)
DANN	0.60 (0.51)	0.35 (0.53)	0.71 (0.85)
Eigen	0.61 (0.55)	0.43 (0.66)	0.77 (0.80)
FATHMM	0.79 (0.83)	0.67 (0.87)	0.85 (0.88)
GERP++	0.57 (0.38)	0.30 (0.38)	0.67 (0.99)
LRT	0.61 (0.49)	0.37 (0.51)	0.71 (0.68)
**MLP**	0.83 (0.68)	0.75 (0.69)	0.89 (0.70)
MetaLR	0.82 (0.83)	0.74 (0.83)	0.88 (0.88)
MetaSVM	0.81 (0.81)	0.72 (0.86)	0.88 (0.87)
MutationAssessor	0.64 (0.73)	0.47 (0.81)	0.78 (0.85)
MutationTaster	0.63 (0.45)	0.41 (0.47)	0.72 (0.80)
PROVEAN	0.63 (0.55)	0.42 (0.60)	0.74 (0.79)
PolyPhen HDIV	0.63 (0.55)	0.42 (0.74)	0.75 (0.88)
PolyPhen HVAR	0.64 (0.59)	0.44 (0.59)	0.75 (0.77)
SIFT	0.64 (0.58)	0.44 (0.66)	0.76 (0.72)
SiPhy 29-way	0.59 (0.44)	0.33 (0.46)	0.67 (0.78)
phastCons 100-way	0.59 (0.39)	0.33 (0.68)	0.67 (0.78)
phyloP 100-way	0.60 (0.51)	0.37 (0.62)	0.73 (0.73)
**sdAE**	0.81 (0.69)	0.72 (0.79)	0.88 (0.79)

### Coverage and missing data

Coverage is another measure of performance: the inability of a score to predict the impact of a subset of SNVs seriously limits its usefulness, hence a good score should have as much coverage as possible. Among the tools we examined, CADD, DANN, GERP++, MutationTaster, phyloP, phastCons and SiPhy demonstrate almost complete coverage of the genome, yet fall short in terms of prediction accuracy. At the same time, the high-performing scores demonstrate significantly limited coverage (Table 1). We designed our scores with high-coverage in mind from the beginning and ran a separate round of tests to evaluate how they perform when other scores fail to predict due to incomplete annotations or other reasons. For each predictor we found unprocessed SNVs in the testing dataset II. If there were more than 50 unpredicted variations, we assessed their impact using our models and calculated the ROC curve AUC scores (Table 5) and the average precision scores (Table 6). Quite surprisingly, our semi-supervised model (sdAE), explicitly designed and trained to reconstruct missing information, performed downright poorly in the absence of predictions made by FATHMM, PROVEAN, MetaLR, MetaSVM and PolyPhen. At the same time, the MLP performs well in the absence of most predictions: it is only sensitive to the absence of FATHMM and PROVEAN scores. Interestingly enough, both the MLP and sdAE predict nsSNVs unprocessed by Eigen with nearly absolute precision. These results show that the MLP not only provides technically extended coverage of the exome but also makes high-quality predictions.

**Table 5:** MLP’s and sdAE’s ROC curve AUC with 95% confidence intervals evaluated on subsets of SNVs from the training dataset II that could not be processed by other predictors.

Score	Missing predictions	MLP	sdAE
Eigen	1175	0.97 (0.94–0.98)	0.95 (0.92–0.97)
FATHMM	898	0.80 (0.75–0.85)	0.34 (0.28–0.42)
LRT	1772	0.94 (0.93–0.95)	0.80 (0.77–0.84)
MetaLR	118	0.76 (0.68–0.85)	0.59 (0.49–0.70)
MetaSVM	118	0.76 (0.67–0.85)	0.59 (0.48–0.69)
MutationAssessor	843	0.90 (0.88–0.92)	0.72 (0.67–0.77)
PROVEAN	426	0.85 (0.81–0.90)	0.47 (0.39–0.55)
PolyPhen HDIV	286	0.85 (0.80–0.89)	0.53 (0.46–0.60)
PolyPhen HVAR	286	0.84 (0.80–0.89)	0.53 (0.45–0.61)
SIFT	514	0.89 (0.85–0.92)	0.59 (0.52–0.66)

**Table 6:** MLP’s and sdAE’s average precision with 95% confidence intervals evaluated on subsets of SNVs from the training dataset II that could not be processed by other predictors.

Score	Number predictions	MLP	sdAE
Eigen	1175	1.00 (0.99–1.00)	0.99 (0.99–1.00)
FATHMM	898	0.52 (0.42–0.62)	0.18 (0.11–0.25)
LRT	1772	0.83 (0.79–0.87)	0.74 (0.69–0.78)
MetaLR	118	0.81 (0.70–0.90)	0.63 (0.51–0.75)
MetaSVM	118	0.81 (0.71–0.90)	0.63 (0.51–0.75)
MutationAssessor	843	0.83 (0.78–0.87)	0.72 (0.67–0.77)
PROVEAN	426	0.69 (0.59–0.78)	0.41 (0.31–0.50)
PolyPhen HDIV	286	0.82 (0.75–0.87)	0.60 (0.52–0.68)
PolyPhen HVAR	286	0.81 (0.74–0.87)	0.60 (0.52–0.68)
SIFT	514	0.79 (0.72–0.86)	0.59 (0.51–0.66)

### GO term enrichment

We measured generalisability in terms of the average prediction success rate across all terms and the number of GO terms significantly enriched in either the subset of correctly classified or misclassified variations (Table 7). Once again, the MLP outperformed other predictors, yielding the highest average success rate. The five highest-scoring predictors (MLP, MetaLR, sdAE, MetaSVM and FATHMM) show identical performance in terms of the number of significantly enriched terms −139, none of which were enriched in the misclassified subset (FDR 5%).

**Table 7:** Average classification success rate across GO terms and the number of significantly enriched terms. The number of terms enriched in the misclassified subset is given in parentheses.

Score	Average success rate	Significantly enriched
**MLP**	0.864	139 (0)
MetaLR	0.857	139 (0)
**sdAE**	0.850	139 (0)
MetaSVM	0.849	139 (0)
FATHMM	0.828	139 (0)
MutationAssessor	0.725	136 (0)
PolyPhen HVAR	0.720	134 (0)
Eigen	0.720	134 (0)
PROVEAN	0.714	133 (1)
SIFT	0.713	134 (1)
PolyPhen HDIV	0.711	135 (0)
CADD	0.707	131 (1)
MutationTaster	0.681	127 (2)
phyloP 100-way	0.671	126 (2)
DANN	0.668	126 (1)
LRT	0.665	122 (3)
SiPhy 29-way	0.642	120 (6)
phastCons 100-way	0.631	115 (10)
GERP++	0.615	111 (14)

Diving deeper into the misclassified subset, we examined term enrichment in its false-positive (FP) and false-negative (FN) sections (Table 8). Here the MLP proved to be the most balanced classifier, that is it doesn’t strongly gravitate towards making neither FN nor FP errors.

**Table 8:** Average deviation of FP/FN rates from equilibrium (imbalance) across all GO terms in the misclassified subsection of the test dataset II and the number of terms significantly enriched in either the FP or FN subsets of the misclassified variations.

Score	Average imbalance	Significantly enriched
**MLP**	0.130	70
FATHMM	0.131	81
PolyPhen HVAR	0.137	93
SIFT	0.138	87
**sdAE**	0.143	82
MetaLR	0.145	78
MetaSVM	0.147	85
PolyPhen HDIV	0.153	100
MutationAssessor	0.161	99
Eigen	0.169	104
PROVEAN	0.170	100
phyloP 100-way	0.183	113
LRT	0.200	116
CADD	0.213	122
DANN	0.223	123
SiPhy 29-way	0.251	128
MutationTaster	0.266	129
phastCons 100-way	0.291	135
GERP++	0.310	136

### Supervised vs. Unsupervised

Some researchers argue that unsupervised inference can help improve prediction quality, which can be hindered by poor coverage of the variome with reliable information on phenotypic categorisation [21]. CADD is probably the most noticeable implementation of this approach. Since multiple studies, including this one, have shown that CADD performs significantly worse than most of its purely supervised rivals, it becomes unclear, whether there is any actual benefit of unsupervised learning in case of nsSNV classification. Most importantly, more complicated tools, such as DANN and Eigen, based on the combination of CADD’s inference model and deep learning, actually performed worse than CADD itself on our tests. Some may argue that this lack of precision is due to the fact that CADD, DANN and Eigen were developed with more attention paid to the variation in noncoding regions. Yet, that doesn’t explain why our own hybrid semi-supervised model, which was absolutely focused on the exome, didn’t beat its purely supervised sibling (though it did outperform most of the other scores we tested). We believe that a lot more research should be invested into unsupervised learning to uncover its full potential (or the lack thereof).

## Conclusion

Here we successfully explored the possibility to efficiently utilise deep learning models to discriminate neutral and likely pathogenic nsSNVs. We tried to use two distinct architectures, one of which made use of unsupervised learning, and optimised hyper-parameters using a genetic algorithm. Although this work was not conceived as a tool comparison, but rather an exploratory study, our results proved that even relatively simple modern neural networks significantly improve prediction accuracy of a deleteriousness prediction tool. Though our semi-supervised model didn’t outperform its purely supervised sibling, it bested most of the scores we tested in the study. Our supervised model showed superior average accuracy as compared to other scores, especially other deep learning-based tools. We have created an open-access web-server so that others could easily access our MLP classifier: http://score.generesearch.ru/services/badmut/. Although this model proved to perform better than any other tool we compared it to in terms of various performance indicators, effective coverage and generalisability, we believe a lot more more should be done to uncover the real potential of unsupervised and semi-supervised models.

## Supporting information

S1 File Supervised training dataset. The final supervised dataset used for training and fine-tuning.
(GZ) Click here for additional data file.

S2 File Additional benchmarks. Multiple additional performance indicators calculated on a range of binary cutoff thresholds.
(TGZ)
